# Iced-Water Enemata in Dysentery

**Published:** 1874-07

**Authors:** 


Iced-Water Enemata in Dysentery.—Dr. B. Wenzel has related in the *Berliner Klinische Wochenschrift* a series of successful cases of dysentery treated by enemata of iced water. They arrested both hemorrhage and tenesmus, and reduced pyrexia; and, after one trial, a patient would call for another enema as soon as the pain recurred. Only rarely was opium given, the treatment being confined to iced water alone. In acute cases he cured. In old chronic cases the benefit was temporary, as in all other modes of treatment. Whilst, therefore, this plan gives relief in chronic cases, Dr. Wenzel concludes that in acute or recent cases it is the most effective at our disposal.—*The Doctor*.

